# Molecular epidemiology of *Streptococcus pneumoniae* isosslated from children with community-acquired pneumonia under 5 years in Chengdu, China

**DOI:** 10.1017/S0950268822001881

**Published:** 2022-12-14

**Authors:** Haojun Chen, Chenggui Liu

**Affiliations:** Department of Laboratory Medicine, Chengdu Women's and Children's Central Hospital, School of Medicine, University of Electronic Science and Technology of China, Chengdu 611731, China

**Keywords:** Children, community-acquired pneumonia, sequence typing, serotype, *Streptococcus pneumoniae*, virulence

## Abstract

*Streptococcus pneumoniae* (*S. pneumoniae*) is one of the most common community-associated pathogens responsible for pneumonia in children. This retrospective study aimed to investigate the molecular characteristics of *S. pneumoniae* isolated from children with community-acquired pneumonia (CAP) under 5 years in Chengdu, China. Molecular characteristics of *S. pneumoniae* included serotype and virulence factor performed by using PCR method and sequence types (STs) determined by sequencing seven housekeeping genes. In addition, the potential relationships between molecular characteristics were depicted by minimum spanning tree and correspondence analysis. The prevailing serotypes were 19F (18.52%), 6B (17.59%), 19A (13.89%), 6A (6.48%) and 23F (5.56%) among 108 isolates. The overall coverage rates of 7-valent, 10-valent, 13-valent, 15-valent and 20-valent pneumococcal conjugate vaccines (PCVs) were 47.32, 48.1, 75, 75 and 78.7%, respectively. Meanwhile, the coverage rates of PCV13 among the isolates from CAP patients aged <1 year were high up to 84.2%. MLST analysis results showed that there were 56 different STs identified, of which the dominant STs were ST271 (22.22%) and ST320 (12.04%). Five international clones among STs were observed, including Spain^23F^‐1, Spain^6B^‐2, Taiwan^19F^‐14, Netherlands^3^‐31 and Denmark^14^‐32. Additionally, most of the isolates carried *ply*, *psaA*, *nanA*, *pavA*, *piaA* and CC271 isolates expressed more of *nanA* than non-CC271 isolates. Moreover, there were strong relevant relationships among STs, serotypes and virulence factors. Considering serotypes and virulence factors together can be used as the foundation for the formulation of vaccine strategy.

## Introduction

Community-acquired pneumonia (CAP) is a common cause of respiratory leading to high mortality in children under 5 years old all over the world, especially in low-income and developing countries. According to the World Health Organization (WHO) report, despite the number of death declining from 920 000 in 2016 to 740 000 in 2019, CAP was still the deadliest infectious disease accounting for 14% of all deaths in children younger than 5 years [1]. The aetiology of CAP is complex constantly caused by various microorganisms, such as bacteria and viruses. It was reported by Ning *et al*. [2] on the aetiology of nearly 100 000 CAP children, *Streptococcus pneumoniae* (*S. pneumoniae*) was ranked top 3 among bacteria isolated from children aged <5 years hospitalised with CAP, indicating pneumococcal pneumonia should not be underestimated.

*S. pneumoniae* possesses a series of virulence factors that are required for immune evasion against the host complement system [3]. Capsular polysaccharide is one of the most vital virulence factors in *S. pneumoniae*, which currently around 100 different capsular serotypes, divided into 25 individual types and 21 serogroups, each composed of several serotypes with related capsulate determinants, have been identified [4]. Pneumococcal conjugate vaccines (PCVs) can enormously decline the incidence of pneumococcal diseases in terms of targeting certain specific capsular polysaccharide of *S. pneumoniae*. For example, after the introduction of PCVs into Shanghai, the potential coverage rate approximately decreased 20% for PCV7, PCV10 and PCV13 [5, 6] in 2018, respectively. The reduction of PCV coverage rates was attributed to increasing vaccinated children. PCV7 was introduced into the Chinese mainland in 2008 and changed to PCV13 in 2017. Regrettably, PCVs were not included in the National Immunization Program. PCV13 vaccination was given only on a voluntary basis. China recommends four doses, including three primary doses (the first dose in 2 months old, every 2 months interval) and one booster dose (in 12–15 months). PCV13 vaccination costs may need to spend one month's income of most people. These reasons lead to a low vaccination rate.

Although it was proved to be effective by vaccination in preventing pneumococcal diseases, the prevalence of CAP remained up to 65.80% in Chinese children under 5 years old because of a low rate of vaccination [7]. In addition, currently available PCVs cannot well dispose of non-PCV serotypes or non-capsular serotypes in isolates [8]. Therefore, many researchers have turned their attention from PCVs to other virulence factors. Well-known virulence factors naturally existed in *S. pneumoniae*, including autolysin (*lytA*), pneumolysin (*ply*), pneumococcal surface adhesin A (*psaA*), pneumococcal surface protein A (*pspA*) [9]. These factors seemed to be highly conserved and widely distributed in human pneumococci, implying the virulence target might be a fantastic vaccine candidate in the future.

Even though currently many studies on the molecular epidemiology of *S. pneumoniae* have been published in China (especially serotype and resistance), little is focused on the underlying relationship among STs, serotypes and virulence factors. So we aimed to investigate the molecular epidemiology of *S. pneumoniae* isolated from children under 5 years with CAP in Chengdu, clarify the relationship between STs, serotypes and virulence factors in order to provide laboratory-related data for pneumococcal infection prevention strategies and vaccines.

## Materials and methods

### Study design

This retrospective study was conducted from January 2019 to January 2021 at Chengdu Women's and Children's Central Hospital, affiliated University of Electronic Science and Technology of China, which has about 2.6 million outpatient visits each year and 1840 establishment beds. The suspected diagnosis of CAP was based on the following symptoms: the onset of systemic infection such as elevated temperature (>38.0 °C) or chills, and suffering from acute lower respiratory tract infection such as cough, gasp, accelerated breathing and abnormal auscultation, which was confirmed by X-ray or computerised tomographic scan of the chest. The eligibility criteria for enrolled participants included the following: (1) diagnosed as CAP (fulfilled above conditions); (2) younger than 5 years old; (3) specimens from patients like nasopharyngeal aspirate, blood or bronchoalveolar lavage fluid isolated *S. pneumoniae* strains; (4) not vaccinated any PCVs. Excluding multiple admissions for the same patient and strains consecutively isolated from the same patient. In addition, hospitalised patient information was retrospectively extracted from the clinical records system, including elementary demographics, clinical manifestation, examination results.

### Specimen collection, culture and identification

Specimens were collected by professional physicians or nurses in accordance with standard operating procedures and then transported as soon as possible to clinical microbiology laboratory, generally which were streaked on 5% sheep blood Columbia agar plate and cultivated overnight at 37 °C in the 5% CO_2_ incubator. Blood specimen was cultured using the BacT/Alert 3D system (BD, US), and then the positive alert blood specimen was transferred and cultured to 5% sheep blood plate. Preliminary identification was performed on basis of morphology and Gram staining of the isolates, subsequently confirmed by VITEK 2 Compact (BioMérieux, France).

### DNA preparation

Firstly sterilised normal saline was used to adjust the suspension to 1 McFarland turbidity. The bacterial suspension was subsequently centrifuged at 10 000 rpm for one minute at 4 °C, and the supernatant was discarded. Ultimately DNA was extracted from *S. pneumoniae* strains using TIANamp Bacteria DNA Kits (TIANGEN, China) according to the manufacturer's instructions.

### Serotyping

Serotyping was determined by multiplex polymerase chain reaction (PCR) as described in the previous article [10]. A total of nineteen primers were assigned to five multiplex reaction systems. A specific primer pair to the *cpsA* was used as the internal positive control. Serotype 6 was divided into 6A, 6B and 6C using the method referred to the previous study [11]. The serotype of isolates belonged to untyped when the serotype was not detected using the method mentioned above. Amplified PCR products were Semi-quantitatively analysed by agarose gel electrophoresis assay, positive bands sequencing was subsequently entrusted to HuaDa Gene Biological Technology Company, Limited, HuaDa, China. Afterward, the coverage rates of PCV were calculated by the proportion of isolates classified serotypes.

### Multilocus sequence typing

Multilocus sequence typing (MLST) was performed by PCR amplification, purification and sequencing of seven housekeeping genes (*aroE*, *gdh*, *gki*, *recP*, *spi*, *xpt* and *ddl*) according to PubMLST database (https://pubmlst.org/spneumoniae/) [12]. Alleles and sequence types (STs) were determined by sequence alignment using MEGA 7.0 software (https://www.megasoftware.net/), next querying MLST database mentioned above. Alleles or STs that were distinct from the current database were submitted to the curator of the database in order to achieve a new number assignment. The STs obtained were compared with Pneumococcal Molecular Epidemiology Network (PMEN) clones (https://www.pneumogen.net/gps/pmen.html). Clonal complex (CC) was defined as a group of STs that shared five or more of seven alleles. Furthermore, the phylogenetic tree based on spliced genetic sequences was constructed by the UPGMA method with 1000-fold bootstrap resamplings and Tamura-Nei substitution model using MEGA 7.0 software. Interactive Tree Of Life (iTOL) v5 (https://itol.embl.de/) was used to display, annotate and embellish the phylogenetic tree [13]. Eventually, we used the goeBURST algorithm that a refinement of the eBURST algorithm by Feil *et al*. [14] implemented in PHYLOViZ 2.0 software (http://www.phyloviz.net/) to display the relationship between the STs and serotypes.

### Virulence genes detection

A total of seven virulence genes were detected using PCR method as previous study described [6], including autolysin (*lytA*), pneumolysin (*ply*), pneumococcal surface adhesin A (*psaA*), pneumococcal surface protein A (*pspA*), neuraminidase (*nanA*), pneumococcal adherence and virulence factor A (*pavA*), ion transporters (*piaA*).

### Statistical analysis

All statistics in this study were performed using SPSS 22.0 software (https://www.ibm.com/analytics/spss-statistics-software). Continuous variables were represented by medians and quartile intervals, and categorical variables were represented by percentage. Categorical data were analysed using a Chi-square test or Fisher's exact test for difference. Correspondence analysis was used to further determine the relationships between STs and serotypes. Two-sided *P*-values <0.05 were considered statistically significant.

## Results

### Demographic and clinical characteristics

A total of 108 children suffering from *S. pneumoniae* CAP were included in this study. Demographic and clinical characteristics from participants were as shown in Table 1. The ratio of male to female was 1.77 (69/39) and their age ranged from 0 to 5 years old, with median age (P25, P75) being 1 (0.75, 2) year. The number of groups based on age was three groups with 38 (35.19%) aged <1 year, 39 (36.11%) aged 1–2 years and 31 (28.70%) aged 2–5 years. The majority of specimen types isolated from the patients were nasopharyngeal aspirate accounting for 97.22% and the rest of specimen types included bronchoalveolar lavage fluid (2, 1.85%) and blood (1, 0.93%).

**Table 1.** Demographic and clinical characteristics from participants

Characteristics	No. of patients	%
Total	108	100
Gender		
Male	69	63.89
Female	39	36.11
Age (years)		
<1	38	35.19
1–2	39	36.11
2–5	31	28.70
People		
Han	103	95.37
Minority	5	4.63
Specimen types from		
Nasopharyngeal aspirate	105	97.22
Bronchoalveolar lavage fluid	2	1.85
Blood	1	0.93
Clinical manifestations		
Fever	76	70.37
Cough	103	95.37
Moist rales	58	53.70
Complications		
Sepsis	14	12.96
Otitis media	5	4.63
Diarrhea	4	3.70
Endobronchitis	2	1.85
Respiratory failure	2	1.85
Length of stay (days; median and IQR)	7 (6–8)	–
Antibiotic use before admission		
Yes	79	73.15
No	29	26.85
Prognosis		
Cured	95	87.96
Improved	13	12.04
Dead	0	0

### Serotypes distribution and vaccine coverage

There were 15 serotypes detected among 100 *S. pneumoniae* isolates in this study, while 8 of all strains were untyped by PCR method. The prevalent serotype was 19F accounting for 18.52%, followed by 6B (17.59%), 19A (13.89%), 6A (6.48%), 23F (5.56%). Classified by age, the predominant serotypes were 19F (21.05%), 6B (20.51%) and 19F (22.58%) among the isolates from CAP patients aged <1 year, aged 1–2 years and aged 2–5 years, respectively.

**Fig. 1.**
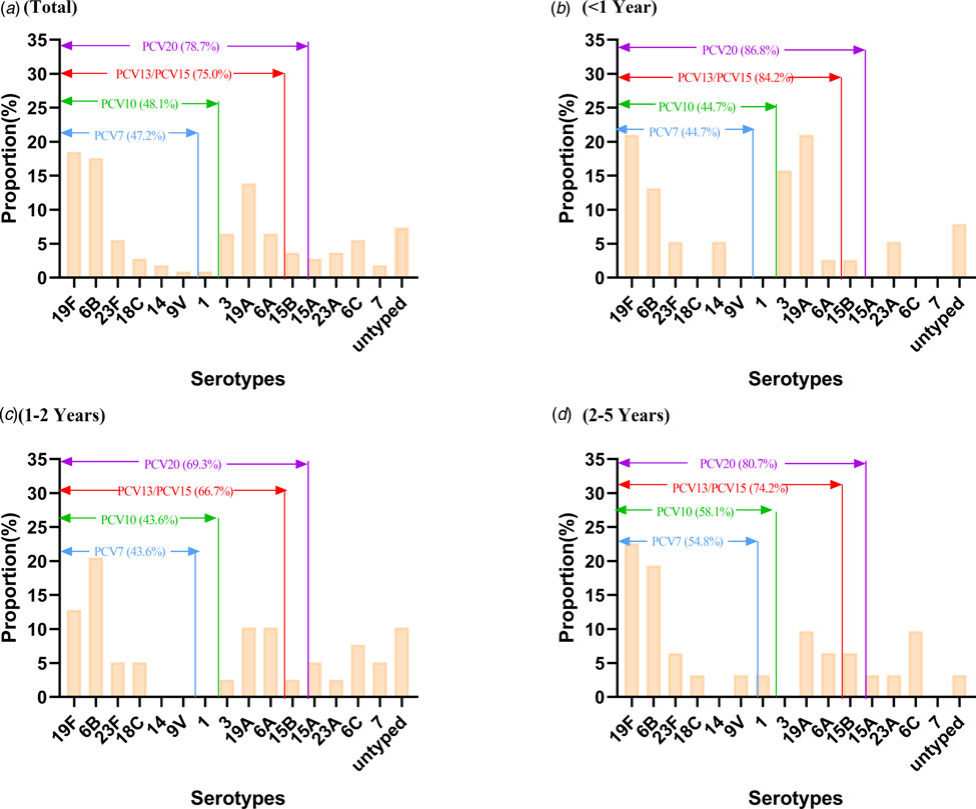
Serotypes distribution and PCVs coverage rates of *S. pneumoniae* classified by ages. (a) All children lower than 5 years; (b) Children under one year; (c) Children aged from one to two years; (d) Children aged from two to five years.

The overall coverage rates of PCV7, 10, 13, 15 and 20 among all isolates were 47.32, 48.1, 75, 75 and 78.7%, respectively (Fig. 1). The coverage rates of PCV7 among the isolates from CAP patients aged <1 year, aged 1–2 years and aged 2–5 years were 44.7, 43.6, 54.8%, respectively, and the coverage rates of PCV13 among those were 84.2, 66.7, 74.2%, respectively, while the coverage rates of PCV20 among those were 86.8, 69.2, 80.6%, respectively. The coverage rates of each PCV among all isolates between different groups sorted by age had no significant difference.

### MLST

**Fig. 2.**
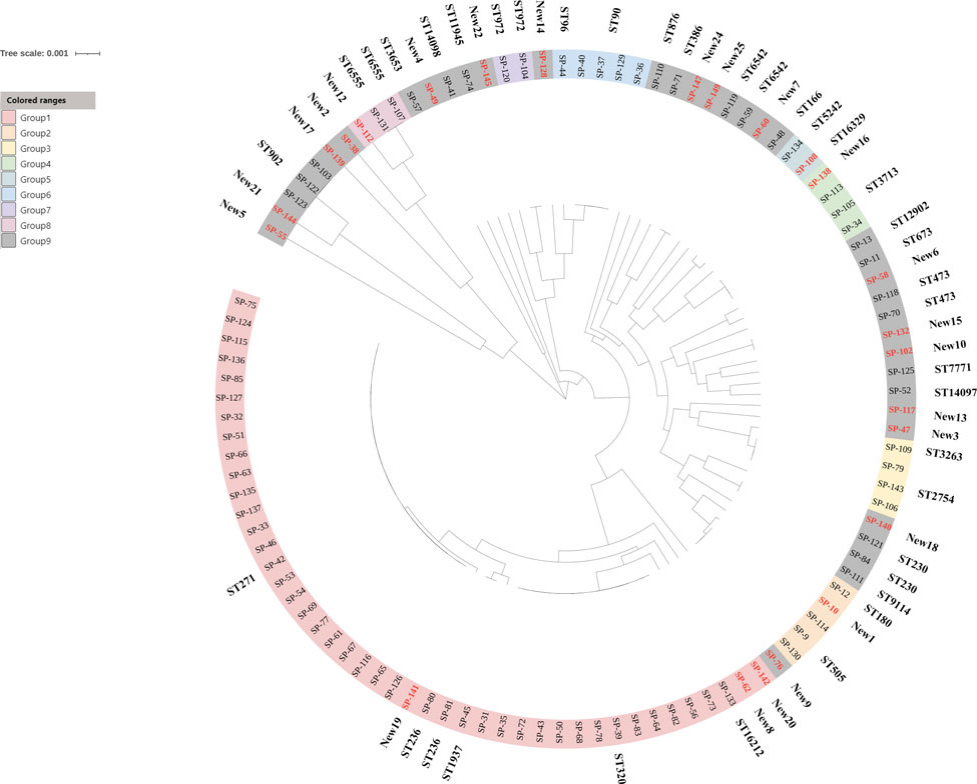
Phylogenetic tree diagram based on concatenated sequences of seven housekeeping genes (*aroE*, *gdh*, *gki*, *recP*, *spi*, *xpt* and *ddl*) of *S. pneumoniae*. ST, sequence type; red bold indicated a novel ST; only 106 isolates except for SP-146 and SP-150 were shown above illustration because of tree scale.

**Fig. 3.**
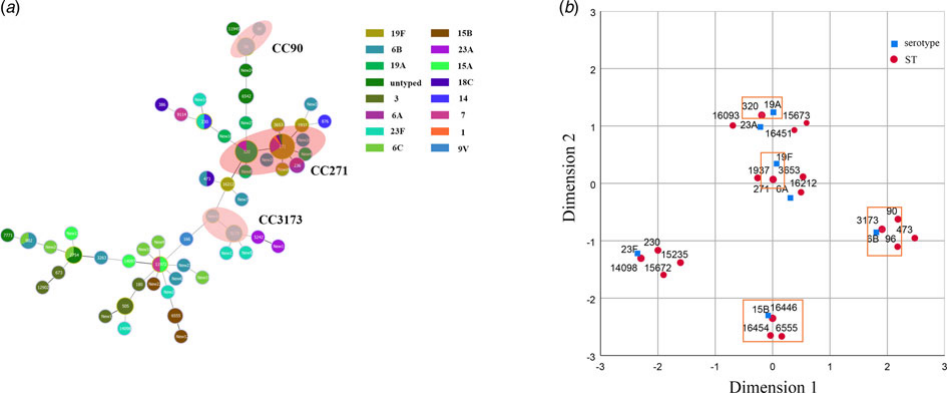
The relationship between STs and serotypes among *S. pneumoniae* isolates. (a) Minimum spanning tree; (b) correspondence analysis.

A total of 56 different STs were identified among 108 *S. pneumoniae* isolates, of which there were 6 novel alleles (*aroE*552-553, *gdh*667, *xpt*937, *xpt*985, *ddl*1015) and 25 novel STs (ST15235, ST15272, ST15665, ST15667-15668, ST15672-15673, ST16093-16094, ST16445-16456, ST16467-16470) found in this study. The dominant STs were ST271 (22.22%) and ST320 (12.04%). As shown in Figure 2, according to the substitution of housekeeping genes nucleotide among these STs, 56 STs were divided into 9 groups defined by bootstrap values of ⩾70%. Every colour strip indicated a CC and the grey strip represented singletons. The prevailing CCs were CC271 (41.67%) with 8 different STs, including ST271, ST320, ST236, ST1937, ST16212, ST16093, ST16452 and ST16468 and CC90 (4.63%) with 2 different STs, including ST90 and ST96. The correlation analysis between STs and serotypes showed that CC271 was closely associated with serotypes 19F and 19A, especially ST271 19F and ST320 19A, accounting for about 25% of all strains. Meanwhile, we also found that the second prevalent serotype 6B was correlated with CC90 (Fig. 3).

A total of five international clones were discovered among 56 different STs in this study, including Spain^23F^‐1, Spain^6B^‐2, Taiwan^19F^‐14, Netherlands^3^‐31 and Denmark^14^‐32. The high prevalence of PMEN clones or their single locus variants (SLVs) were Taiwan^19F^‐14 and Spain^6B^‐2 accounting for 14.81%, 4.63% respectively. Netherlands^3^‐31 was also identified in this study and this group consist of two double locus variants (DLVs) with ST505 (*n* = 3) and ST15272 (*n* = 1) (Supplementary Material).

### Virulence genes distribution and pattern

Virulence genes distribution was listed in Table 2. Generally, all isolates carried *lytA* and most of the isolates carried *ply* (99.07%), *psaA* (98.15%), *nanA* (88.89%), *pavA* (96.30%) and *piaA* (97.22%). Approximately 45.37% of the isolates harboured *pspA*. It was worth noting that parts of virulence factors were significantly related to CC. In brief, the carriage rate of *nanA* in the CC271 group was higher than that in the non-CC271 group (*P* = 0.013), inversely the carriage rate of *pspA* in the CC271 group was lower than that in the non-CC271 group (*P* < 0.001).

**Table 2.** Virulence genes distribution of *S. pneumoniae* isolated from children with CAP

Virulence genes	Total *n* (%) (*n* = 108)		CC271 *n* (%) (*n* = 45)		Non-CC271 *n* (%) (*n* = 63)		*χ*^2^	*P*
	Positive	Negative	Positive	Negative	Positive	Negative		
*lytA*	108 (100)	0 (0)	45 (100)	0 (0)	63 (100)	0 (0)	–	–
*ply*	107 (99.07)	1 (0.93)	44 (97.78)	1 (2.22)	63 (100)	0 (0)	1.413	0.235
*psaA*	106 (98.15)	2 (1.85)	45 (100)	0 (0)	61 (96.83)	2 (3.17)	1.456	0.228
*pspA*	49 (45.37)	59 (54.63)	1 (2.22)	44 (97.78)	48 (76.19)	15 (23.81)	57.945	**<0**.**001**
*nanA*	96 (88.89)	12 (11.11)	44 (97.78)	1 (2.22)	52 (82.54)	11 (17.46)	6.171	**0**.**013**
*pavA*	104 (96.30)	4 (3.70)	42 (93.33)	3 (6.67)	62 (98.41)	1 (1.59)	1.899	0.168
*piaA*	105 (97.22)	3 (2.78)	42 (93.33)	3 (6.67)	63 (100)	0 (0)	2.204	0.138

CAP, community acquired pneumonia.

Bold indicated statistical significance among different CC groups (*P* < 0.05).

There were 11 virulence patterns found in this study. The most common patterns were *lytA*-*ply*-*psaA*-*nanA*-*pavA*-*piaA* profile among all isolates accounting for 46.30%, with the relevant serotype being 19F and 19A and the *lytA*-*ply*-*psaA*-*pspA*-*nanA*-*pavA*-*piaA* profile among the isolates accounting for 35.19%, with correlated serotype being 6B (Table 3).

**Table 3.** The relationship between virulence patterns and serotypes of *S. pneumoniae* isolated from children with CAP

Virulence pattern	Isolates (No.)	Proportion (%)	Related serotypes (No.)
*lytA-ply-psaA-pspA-nanA-pavA-piaA*	38	35.19	6B (16), 3 (6), 23F (1), 15A (2), 6C (3), 7 (1), 23A (2), 15B (1), 18 (2), untyped (4)
*lytA-ply-psaA-pspA-nanA-piaA*	1	0.93	19F (1)
*lytA-ply-psaA-nanA-piaA*	2	1.85	19F (1), 23F (1)
*lytA-ply-psaA-piaA*	1	0.93	1 (1)
*lytA-ply-psaA-nanA-pavA*	2	1.85	19F (1), 19A (1)
*lytA-psaA -nanA-pavA*	1	0.93	19F (1)
*lytA-ply-psaA-pspA-pavA-piaA*	9	8.33	14 (1), 6C (2), 23F (3), 6B (2), untyped (1)
*lytA-ply-psaA-pavA-piaA*	2	1.85	14 (1), 7 (1)
*lytA-ply-nanA-pavA-piaA*	1	0.93	23F (1)
*lytA-ply-pspA-nanA-pavA-piaA*	1	0.93	untyped (1)
*lytA-ply-psaA-nanA-pavA-piaA*	50	46.30	19F (16), 19A (14), 15 A(1), 15B (3), 18B/C (1), 23A (2), 6A (7), 6B (1), 6C (1), 9V (1), 3 (1), untyped (2)

CAP, community acquired pneumonia.

## Discussion

*S. pneumoniae* is one of the common pathogens in CAP that leading cause of mortality in children under 5 years old. To prevent pneumococcal-related diseases and reduce the economic burden of that, many developed countries have introduced PCVs targeting serotypes of *S. pneumoniae* into National Immunization Program. However, PCVs targeting capsular polysaccharide locus cannot overcome replacement serotype or non-capsular serotypes among *S. pneumoniae*. Virulence factors, as good antigen targets, can effectively supplement the failure of PCVs. Therefore, this study aimed to investigate molecular characteristics of STs, serotypes and virulence factors of *S. pneumoniae* isolated from children less than 5 years with CAP in Chengdu to provide data support for development of vaccines and formulation of pneumococcal infection prevention strategies.

Large, accurate, long-term serotype distribution monitoring of *S. pneumoniae* isolates contributes to better multivalent vaccine development and guides for vaccination. In this study, the prevalent serotype was 19F, followed by 6B, 19A, 6A, 23F, which resembled other cities in China or Asian countries, but their rank order varied by region [5, 15, 16]. However, the prevailing serotypes were 19A, 7F, 3 and 1 in Europe and that was 14, 1 and 5 in America [17, 18]. Exception for region factor, the distribution of *S. pneumoniae* serotypes was limited by vaccine usage, age and so on. For example, the prevalent serotypes were 19F, 4, 6, 18 in the pre-PCV7 period in Russian children, and that serotypes were 4, 6, 18 after the introduction of PCV7 [19], indicating a substantial reduction of 19F by vaccination. It also can be seen that different multivalent vaccines can only prevent infection with certain serotypes of *S*. *pneumoniae*. In this study, the overall coverage rates of PCV7, 10 among all isolates were 47.32%, 48.1%, respectively. PCV10 seemed not better than PCV7 because 1, 5, 7F were rarely detected in our study. When taking considering into age factor, we found that the coverage rate of PCV13, PCV15 and PCV20 among isolates from children aged <1 year was as high as 84.2, 84.2 and 86.8%, respectively, which surpassed that of PCV7 and PCV10 among isolates from all children with no vaccination. We strongly suggest that it would be best to vaccinate with PCV13 for infants in the high coverage areas of serotypes, which can release the pressure caused by most vaccine-covered pneumococcal diseases.

Compared to pulsed-field gel electrophoresis technology, MLST is a simple, effective, comparable method to describe the molecular characterisation of microorganisms. It has been applied in many aspects, such as epidemiological investigation, molecular evolution of bacteria [20]. In this study, CC271 was the most common group of STs, including the prevalent ST271 and ST320. A few previous research studies have suggested that there is a good correlation between STs and serotypes. For example, ST320 19A was reported as the prevalent clone in Latin American [21], while ST271 19F was prevalent in China [22]. By using minimum spanning tree analysis, we found that ST271 19F and ST320 19A originated from Taiwan^19F^‐14 were common clones in this study. Except for two common international clones Taiwan^19F^‐14 and Spain^6B^‐2 in Chengdu, two DLVs of Netherlands^3^‐31 (ST505 and newly ST15272) were founded in this study. The two DLVs belonged to new clones in China, which may be related to the worldwide mainstream CC180 [23]. Additionally, there may exist also consistency between some STs and serotypes due to the limited sample size in this study, such as ST505 with serotype 3, ST3173 with serotype 6B, ST876 with serotype 14, which was similar to the previous studies reported in China [9, 23]. Interestingly, one ST may contain diverse serotypes sometimes. In this study, ST271 included 6A and 1 besides the absolutely prevailing 19F. The results by Tocheva *et al*. [24] also showed that ST199 contained serotypes 19A, 14, 15 and ST162 contained 19F, 9V. Taken together, these results of studies indicated there was the evolution of serotypes switching among STs in order to adapt vaccine or antimicrobial pressure [25].

It is established that virulence factors, as important components of bacteria, can be alternative vaccine antigens. Recent report proved that conserved proteins that have been proposed for candidate vaccines should possess favourable immunogenicity and immunoreactivity of antigen, such as *pspA*, inhibiting the activation of the C3 complement protein and elevating the colonisation ability of *S. pneumoniae* [26]. Besides the above-mentioned conditions, an additional requirement for selecting virulence factors as candidate vaccines is widespread distribution in the population. In this study, all isolates carried *lytA* and most of the isolates carried *ply*, *psaA*, *nanA*, *pavA* and *piaA*, which was similar to other cities of China [6]. Based on our results, we suggested that these virulence factors including *lytA*, *ply*, *pasA*, *nanA*, *pavA* and *piaA* might be candidate vaccines in the future. However, the carrier rate of *pspA* was significantly lower than that of other virulence factors, which might be not suitable for vaccine candidate. Currently, little is known about the relationship between ST/CC and virulence factor. A few previous studies reported that CC271 carried more virulence factors than non-CC271 [27, 28]. In this study, the positive rate of *nanA* in the CC271 was higher than that in the non-CC271. Recent research has revealed that pneumococcal neuraminidase A encoded by *nanA* gene can synergise with that of influenza A virus strengthening cytotoxicity [29]. These results together indicated CC271 harbouring more *nanA* might exhibit much pathogenicity. Additionally, the most common virulence pattern was *lytA*-*ply*-*psaA*-*nanA*-*pavA*-*piaA* mainly distributed in serotype 19F and 19A in this study, while *lytA*-*ply*-*psaA*-*pspA*-*nanA*-*pavA*-*piaA* pattern in serotype 6B. It can be seen that the distribution of virulence factors might be associated with serotypes. Virulence factor *pspA* consisted of two families (family 1 and family 2), whose distribution was different from region. It was reported that the carriage rate of family 2 was higher than that of family 1 in Japan [30]. Although *pspA* of 19F and 6B belong to family 1, their distribution is also distinct. Our results indicated that serotype 6B isolates were more aggressive than 19F isolates. Furthermore, purified protein-vaccine from the homologous family 2 cannot against infection caused by *S. pneumoniae* carrying family 1 [31, 32]. Therefore, we considered that supplemental vaccine strategy that PCVs coupled with certain virulence factors maybe reduce the burden of pneumococcal disease based on the distribution of STs, serotypes and virulence factors [33].

To the best of our knowledge, this is the first study on potential relationships between molecular characteristic of *S. pneumoniae* from children with CAP under 5 years in Chengdu via minimum spanning tree and correspondence analysis, while several limitations warrant attention in this study. Firstly, the sample size of pneumococcus needs to be further expanded to a more comprehensive assessment of molecular epidemiology of *S. pneumoniae*. Secondly, some serotypes have not been identified so far, which might need other methods to identify. Ultimately, the results only represent the data of the last two years when the time span of specimens collection was between January 2019 and January 2021 in this article. More high-quality and long-term surveillance on STs, serotypes, virulence factors needs to be carried out in the future.

In summary, our findings indicated that CC271 was the predominant epidemic clone, especially ST271 19F and ST320 19A. Moreover, there were strong relationships between STs, serotypes, virulence factors, which indicated the formulation of vaccine strategies needs to carefully consider molecular epidemiological factors.

## Data Availability

All datasets generated for this study are included in the manuscript.

## Supplementary material

For supplementary material accompanying this paper visit http://doi.org/10.1017/S0950268822001881. click here to view supplementary material
