# Molecular epidemiology of *Streptococcus pneumoniae* isolated from children with community-acquired pneumonia under 5 years in Chengdu, China – CORRIGENDUM

**DOI:** 10.1017/S0950268823000080

**Published:** 2023-02-02

**Authors:** Haojun Chen, Chenggui Liu

**Affiliations:** Department of Laboratory Medicine, Chengdu Women's and Children's Central Hospital, School of Medicine, University of Electronic Science and Technology of China, Chengdu 611731, China

The word in the title ‘isolated’ was initially misspelled as ‘issosslated’. The typo has now been corrected retrospectively both in the article proof and the HTML.

The author and the publisher apologize for the error.

## References

[ref1] Chen, H., & Liu, C. (2023). Molecular epidemiology of Streptococcus pneumoniae isosslated from children with community-acquired pneumonia under 5 years in Chengdu, China. Epidemiology and Infection, 151, E2. doi:10.1017/S0950268822001881 PMC9990402 36515066

