# Supplementary figures and images for: Molecular epidemiology of Streptococcus pneumoniae isosslated from children with community-acquired pneumonia under 5 years in Chengdu, China
Source: Epidemiol Infect. 2022 Dec 14;151:e2. doi: 10.1017/S0950268822001881 (PMC9990402; doi:10.1017/S0950268822001881)

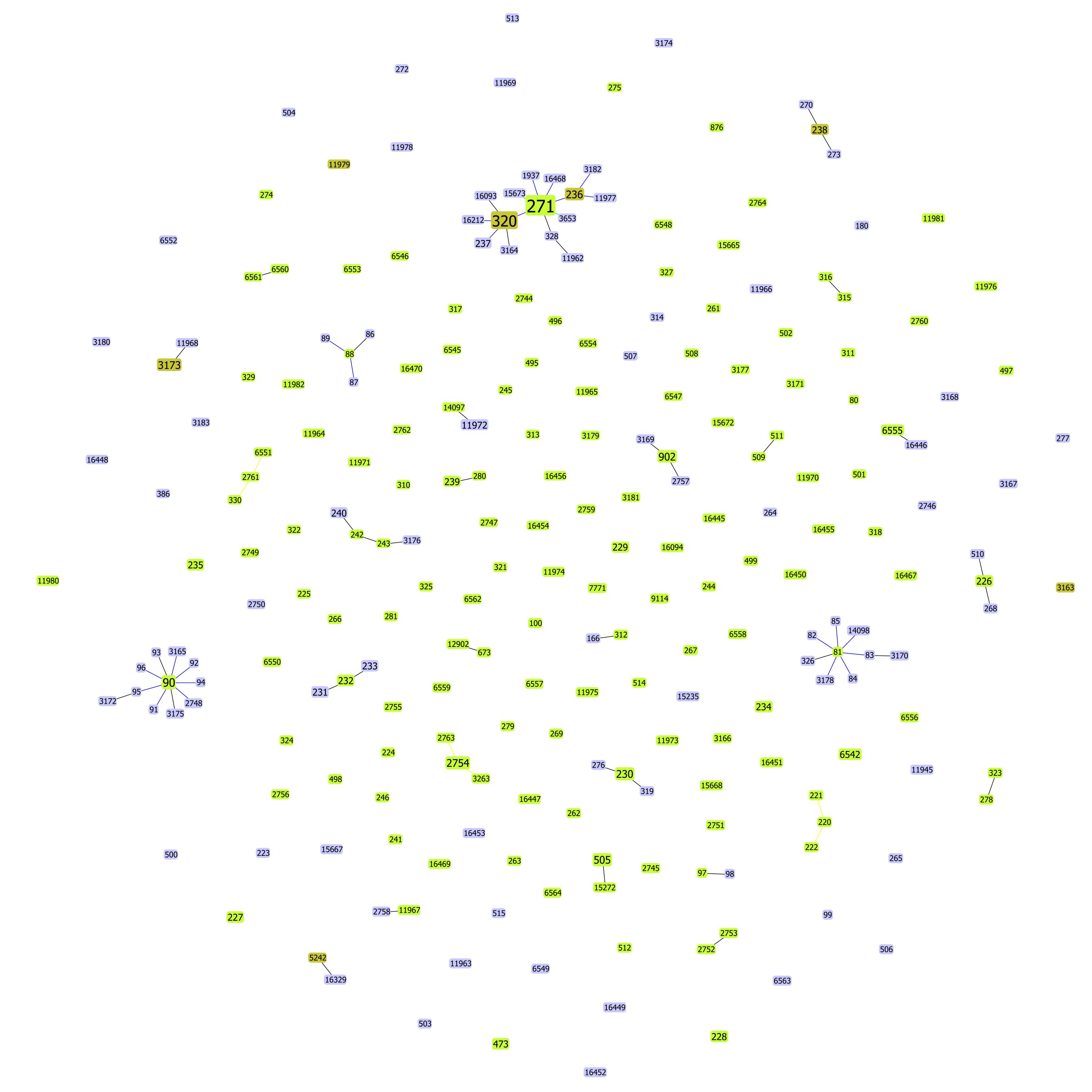

Supplement: Supplementary file 1 [file hygsup.zip › S0950268822001881sup001.tiff]
